# System-based approaches as prognostic tools for glioblastoma

**DOI:** 10.1186/s12885-019-6280-2

**Published:** 2019-11-12

**Authors:** Manuela Salvucci, Zaitun Zakaria, Steven Carberry, Amanda Tivnan, Volker Seifert, Donat Kögel, Brona M. Murphy, Jochen H. M. Prehn

**Affiliations:** 1 Centre for Systems Medicine, Department of Physiology and Medical Physics, Royal College of Surgeons in Ireland, 123 St Stephen’s Green, Dublin 2, Ireland; 2 Department of Neurosurgery, Frankfurt University Hospital, Frankfurt am Main, Germany

**Keywords:** Apoptosis, Computational model, Glioblastoma, Molecular signatures, Network model, Numerical simulation, Precision oncology, Prognostic biomarker, Systems biology, Systems medicine

## Abstract

**Background:**

The evasion of apoptosis is a hallmark of cancer. Understanding this process holistically and overcoming apoptosis resistance is a goal of many research teams in order to develop better treatment options for cancer patients. Efforts are also ongoing to personalize the treatment of patients. Strategies to confirm the therapeutic efficacy of current treatments or indeed to identify potential novel additional options would be extremely beneficial to both clinicians and patients. In the past few years, system medicine approaches have been developed that model the biochemical pathways of apoptosis. These systems tools incorporate and analyse the complex biological networks involved. For their successful integration into clinical practice, it is mandatory to integrate systems approaches with routine clinical and histopathological practice to deliver personalized care for patients.

**Results:**

We review here the development of system medicine approaches that model apoptosis for the treatment of cancer with a specific emphasis on the aggressive brain cancer, glioblastoma.

**Conclusions:**

We discuss the current understanding in the field and present new approaches that highlight the potential of system medicine approaches to influence how glioblastoma is diagnosed and treated in the future.

## Background

Systems biology combines computational technology, numerical techniques and wet-lab research to disentangle and simulate complex biological networks, allowing researchers to understand a system’s processes and mechanisms, deliver novel therapeutic targets, and stratify patients for clinical trials [1–5]. Systems biology approaches have been successfully applied across multiple fields of biomedical sciences including immunology [6], inflammation [7], cardiology [8], sepsis [9], respiratory distress [10], neurodegenerative disorders [11] and cancer [12]. The arrival of cost-effective, high throughput -*omic* profiling and imaging technologies, in combination with advances in mathematical modelling, bioinformatics and machine learning, has initiated translational studies that apply, for the first-time, systems biology approaches in the clinic [12–24]. Indeed, such approaches often require larger multi-disciplinary teams of in silico researchers, biologists and clinician scientists for tool development, resourcing of material to be investigated and interpretation of data.

In this review, we will outline how systems biology has been instrumental in advancing precision oncology encompassing early detection, diagnosis monitoring and treatment, focusing on glioblastoma multiforme (GBM).

## Main text

### Glioblastoma in the era of systems biology

Glioblastoma is a grade IV primary brain tumor and the most aggressive form of all types of glioma, with a median survival of 12–15 months [25, 26]. GBMs may be classified into two categories, based on the history of tumor onset. Newly-diagnosed GBMs represent more than 90% of GBMs, clinically presenting as de novo cases, while the remaining 10% of tumors are classified as secondary GBMs progressing from low-grade (grades I–III) gliomas. As both are highly diffuse and invasive by nature, it is not possible to achieve complete surgical resection of all tumor cells [27]. Therefore, cytotoxic insults with temozolomide (TMZ) chemotherapy and radiotherapy are the first-line adjuvant therapy delivered [26] to inhibit cell proliferation and trigger apoptotic cell death [17, 28]. Despite great strides in targeted therapeutics in both pre-clinical and clinical studies, an exceptionally high fraction of GBM patients experience recurrence and prognosis remains extremely poor [29–31]. Moreover, current standard-of-care for both tumors resected at initial diagnosis and at recurrence is largely based on study performed on samples from newly-diagnosed patients.

To date there is an extremely limited availability of molecular markers for prognostic and predictive markers with clear clinical relevance [32]. Treatment of the disease is largely ‘one size fits all’, even though GBM patients that are female, with methylated O^6^-methylguanine-DNA methyltransferase (MGMT) promoter, younger age, who underwent resection rather than biopsy and with pre-operative Karnofsky performance score ≥ 70 are associated with better outcome [33–37].

The development of any form of cancer is an extremely complex process involving the acquired mutation of multiple independent genes, engaging novel genes and signaling pathways that alter cell proliferation, cell growth, bioenergetics, apoptosis sensitivity, angiogenesis and immune evasion, among many others [38, 39]. Because of this complexity, application of systems biology via both data- and hypothesis-driven approaches for novel diagnostic and prognostic techniques for oncology treatment is an advancing research niche [13, 15–21, 40–48].

### Perspective on data-driven biomarkers

Recent technological advances coupled with unprecedented computational infrastructures have provided researchers with large-scale data from multiple sources that can be harnessed to improve cancer detection, diagnosis and treatment. A major source of data include imaging studies such as scans from computer tomography (CT) and magnetic resonance (MRI) [49, 50], and microscopic pathology [51–55] routinely performed for diagnosis and assessment of treatment response. *Omic* data, such as mutations and expression of genes and proteins [42, 43, 56–63] derived from samples from blood or tumor tissue from biopsy or surgical resection are another fertile source of biomarkers for the disease. More recently, data extracted from electronic medical records [64, 65], mobile health applications [66, 67], social media [68, 69] and web searches [70] have also been harnessed. Hybrid approaches leveraging data from multiple data types have also emerged as promising biomarkers [71–73].

A major focus of GBM research over the past decade has been determining and understanding the molecular architecture of GBM predominantly from a genomic, epigenomic and transcriptomic standpoint. Through the efforts of the Cancer Genome Atlas (TCGA) [74] and other consortia, between two to four GBM subtypes have been classified based on transcriptomic profiling: proneural (PN) and mesenchymal (MES) have been most reliably established, with classical (CL) and neural subtypes also described [43, 62, 75]. The PN subtype arises in the frontal cortex of younger patients, accompanied by platelet-derived growth factor receptor-A (PDGFRA) amplification [43, 76], isocitrate dehydrogenase (IDH) 1/2 mutation [42, 43, 77, 78], and tumor protein p53 (TP53) mutations [43, 79, 80]. Patients harboring IDH1/IDH2 mutations and often a CpG island methylator phenotype (G-CIMP) are constituted for the vast majority by secondary GBMs and have the best prognosis of any GBM subgroup [43, 81]. In contrast, proneural GBM patients with wild type IDH status have a significantly worse outcome in terms of progression-free survival (PFS) rates [43, 78]. MES subtype of GBM is an extremely aggressive form, with greater vascularity [43, 82], and an associated with neurofibromin (NF1) lesions [43, 83, 84], nuclear factor kappa-light-chain-enhancer of activated B cells (NF-κB) activity [43, 85, 86], increased expression of protein kinase B (PKB or AKT), and tumor growth factor-beta (TGF-β) [43, 87, 88]. The CL subtype is also aggressive, distinguished by the presence of epidermal growth factor receptor (EGFR) lesions [43, 89]. The neural subtype however has been debated and has become controversial, due to the fact that it is less distinct and is thought perhaps to arise from possible contamination of GBM samples with normal brain tissue [43, 62].

Recent work by Suvà laboratory [90] integrating bulk and single-cell transcriptomics from adult and pediatric GBM tumors with lineage tracing experiments and patient-derived xenograft (PDX) models has shown how GBM cells can assume four distinct cellular states ranging from neural-progenitor-like; oligodendrocyte-progenitor-like; astrocyte-like and mesenchymal-like. While both adult and pediatric tumor samples contain a mixture of 2 to 4 of these cell states (with astrocyte-like being under-represented in pediatric), their proportion reflects the genetics and transcriptomic program of the tumors. Tumor cell can transition between states and this plasticity is a function of their genetic, epigenetic and tumor micro-environment make-up and it is mirrored in their transcriptional subtype. Cells with an astrocyte-like state feature EGFR aberrations and are found in tumors with a CL transcriptomic subtype. Similarly, cells with a mesenchymal-like state present with aberrations in NF1 and chromosome 5q (Chr5q) deletions. In contrast, the PN subtype includes a mixture of both neural-progenitor-like (Cyclin-dependent kinase A (CDKA) amplifications) and oligodendrocyte-progenitor-like (platelet-derived growth factor receptor-A (PDGFRA) aberrations).

While the utilization of such tumor classification based on transcriptomic subtypes is not yet widespread in the clinic, it is hoped that through identification and understanding of critical drivers of each GBM subtype, this will lead to more personalized therapeutic approaches for patients and improved survival rates [42, 43, 62, 74, 79]. A significant caveat of this approach with regards to GBM tumors, however is that potential biological targets which are identified based on the biology of the first GBM tumor may not be present in the recurrent tumor [62, 75, 91–93]. Indeed, recent research has put forward the notion that multiple longitudinal specimens sampled from spatially-distinct regions of the tumor are necessary to characterize continuously evolving and high heterogeneous GBMs [94–97]. Re-characterization of the isolated tissue should be performed, especially in settings where targeted agents will be employed, to ensure the targets are relevant for a sizable fraction of the tumor cells and still present at recurrence [62, 92, 98]. GBM notorious intra-tumor heterogeneity translates into the presence of multiple transcriptomic subtypes within a patient tumor [62, 99, 100]. Moreover, the expression of the transcriptomic programs differs between contrast-enhancing and non-contrast-enhancing regions of the tumor [101]. Wang et al. found that approximately two thirds of recurrent samples have a different (dominant) transcriptomic subtype at recurrence compared to their primary tumor, with the MES program emerging as the most stable [98]. The transition from PN to MES transcriptomic program (analogous to epithelial to mesenchymal transition) [102] features among the marked molecular differences identified from genomic, epigenomic and transcriptomic analyses in tumor samples from newly-diagnosed and recurrent patients (recently reviewed in [92, 103]). Recent research has focused on developing treatments specific to transcriptomic subtyping. Anti-angiogenic treatment has emerged as treatment of choice for patients with MES tumors as morphometric analyses have shown that these tumors have larger, but not more numerous, vessels with larger necrotic and hypoxic areas. Disappointingly, a clinical trial did not reveal survival benefit when Bevacizumab was administered in combination with lomustine to GBM patients not selected based on transcriptomic subtype [104].

### Perspective on hypothesis-driven biomarkers

Dynamic systems modelling techniques, often based on ordinary differential equations, can be used to understand the complex and often nonlinear relationship between multiple components within a biological system. Such systems models calculate the dynamic changes in the various elements of signal transduction pathways and take into account network topology and biochemical pathways including feed-back/forward loops and alternative pathway branches. Because of their quantitative nature, dynamic systems models can also be employed to qualitatively and quantitatively predict responses to therapies that target the signaling network under investigation [105–107]. Such modelling techniques have been successfully employed in the case of apoptosis (reviewed in [108]), kinase [109–111] and microRNA signaling [112]. Dynamic systems models have the potential to deliver powerful prognostic biomarkers for the clinical management of cancer that outperform statistical approaches, and have been shown to significantly improve ‘traditional’ histopathological risk factors of disease progression [12, 15–22, 44, 47].

Work from the Swanson lab has highlighted how relevant parameters can be extracted from MRI scans routinely performed as part of the diagnostic and treatment monitoring protocol and how they can be leveraged as input to a mathematical model dabbed “Proliferation-Invasion” to estimate the nature and aggressiveness of GBM tumors. This work builds upon the pioneering work of Murray [113] and models the “Go or Grow” nature of cancer with a spatio-temporal framework where cells either migrate (go) or proliferate on site (grow). Swanson et al. detailed how net migration and proliferation rates can be estimated from macroscopical features of the tumor detected in MRI scans on a patient by patient basis and demonstrated how patients with nodular tumors (low migration and high proliferation) have better survival prospects compared to those with diffuse (high migration low proliferation) tumors. By extracting the parameters from pre- and post-treatment MRI scans, the model leads to the calculation of “days gained” as a proxy for treatment success. Patients predicted to benefit from the treatment (higher days gained) exhibited significantly longer PFS and overall survival (OS). These results suggest that, if implemented in clinical practice, days gained could be used to monitor patient response to treatment and to identify promptly case with insufficient response requiring an alternative therapeutic regimen [15, 16].

Interestingly, this modelling approach has been recently applied in conjunction with other data-driven approaches [37, 114] highlighting how these two branches of systems biology can be complementary and integrated.

Yang et al. applied this class of models in support of data-driven analysis in the investigation of differences in transcriptomic programs between male and female and their putative role in treatment response and outcome [37]. Initial tumor growth velocities predicted by the mechanistic model from serial MRI scans showed no difference between male and female subjects. In contrast, the authors observed a decrease in velocities following TMZ treatment in female, but not male subjects. When comparing survival curves from females with high vs. low predicted first TMZ velocities, the authors found that females with low velocities survived longer. These associations were null in male patients [37]. Moreover, gene expression analysis identified transcriptomic programs common to both sex (calcium-calmodulin signaling) along with pathways specific to male (cell cycles signaling) and females (integrin signaling). Interestingly, Yang et al. also observed a differential role played by the IDH mutation and transcriptional subtypes by sex.

Gaw and co-workers [114] integrated the mechanistic insights from the “Proliferation-Invasion” model (PI) with feature extractions capabilities by semi-supervised machine learning (ML) into a hybrid model (dabbed ML-PI). The ML-PI model takes as input images from sequential MRI scans and outputs a (spatial) map of tumor cells that can help clinicians identify the invasive front of the tumor (under-estimated by current MRI enhancement signal) aiding in planning radiation treatment and, in the future, surgery. In this proof-of-concept study performed on MRI scans from *n* = 18 newly-diagnosed GBM patients the authors found improved performance when using the hybrid model (ML-PI) compared to either single algorithm (ML or PI) highlighting the importance of leveraging both the data- and hypothesis-driven branches of systems biology to advance precision oncology [114].

Fey et al. developed an ODEs system for the JUN N-terminal kinase (JNK) pathway by applying rule-based modelling in tandem with extensive in vitro validation (including SH-SY5Y cells). The model takes as input the kinase expression for ZAK (sterile alpha motif and leucine zipper containing kinase), AKT, MKK4 (dual specificity mitogen-activated protein kinase kinase 4), MKK7 (dual specificity mitogen-activated protein kinase kinase 7) and JNK, either protein- or transcriptomic-based, and predicts JNK activation via phosphorylation. The model can satisfactory reproduce experimental profiles of JNK activation mediated by isomycin and other stressors. Importantly, features extracted from the model-predicted JNK activation profile, namely signal amplitude, half-activation threshold and, particularly, ultrasensitivity (i.e. Hill coefficient) were identified as control points and potential treatment avenues. The model development was geared towards the neuroblastoma settings, a type of childhood cancer with remarkably diverse prognosis ranging from spontaneous remission to death. Patients with N-Myc (MYCN) amplification (~ 20–25% of the cases) have worse prognosis, however poor outcome is observed also in children not affected by this molecular change. Fey and colleagues described how patients whose simulations showed a higher amplitude and a more marked switch-like behavior (higher Hill coefficient) in JNK activation, suggestive of a functional apoptosis machinery, had better outcome compared to those with a more dampened response. Remarkably, the model prognostic value was demonstrated in a training and two validation cohorts (over 700 patients) and found to be independent of MYCN amplification status.

By combining mathematical modelling with carefully designed experiments in patient samples, cell-line and patient-derived xenografts and patient-derived cell cultures, Niclou’s laboratory has recently recapitulated phenotypic plasticity [115]. This research work put forward the notion that cancer stem cells (CSCs) do not represent a separate class of cells with immutable features. In contrast, this work indicates that the microenviroment may mediate the (reversible) transition from a non-stem cell to a stem cell-like phenotype. The authors describe transitions across cell states as a Markovian process depending on the current cell state and microenviroment signals (drawn from normoxia/hypoxia and in vivo scenarios). Results from computational and experimental analyses revealed that the degree of plasticity was associated with tumorigenesis potential in the in vivo settings and recommend that future therapeutic endeavors should focus on harnessing this plasticity as opposed to targeting the cancer stem cells subpopulation [116].

Chemo- and radiation-therapy require functional apoptosis pathways to be effective. Indeed, apoptosis deficiency, induced by an imbalance among pro- and anti-apoptotic agents, characterizes the proteomic landscape of the vast majority of tumors and is the object of a vast body of research in GBM [17, 117–122].

#### Apoptosis systems modelling in oncology

Systems biology tools describing the ‘all-or-none’ nature of apoptosis signaling have been developed over several years through a close interaction of in silico and wet lab-based research [47, 123–126]. The application of systems modeling for apoptosis research began in 2000 by Fussenegger et al., [127] focusing on mathematical models with ordinary differential equations (ODEs). Fussenegger et al. recapitulated into a mathematical framework how initiator caspases from both the intrinsic and extrinsic pathways, when triggered, activate effector caspases leading to apoptosis. The model includes activation of initiator caspases 8 and 9 leading to the formation of the apoptosome and death-inducing signaling complexes via stress- and receptor-mediated mechanisms, respectively. Inhibition mechanisms for initiator caspases by anti-apoptotic proteins from the B-cell lymphoma 2 (BCL-2) family (BCL-2 and B-cell lymphoma-extra large (BCL-xL)) and decoy receptors and by inhibitor of apoptosis (IAPs) proteins for executioner caspases, respectively, are also modelled. The authors demonstrated that the model output (active caspases) was in agreement with experimental results under different simulation scenarios and could be used to simulate perturbation by therapeutic interventions (disruption of Fas-associated protein with death domain (FADD), BCL-2 or inhibitor of apoptosis (IAP) overexpression) to promote or inhibit cell death. Our understanding of the apoptosis pathways has progressed dramatically and mathematical models have followed suit, as outlined in Table 1, providing valuable insight into the mechanistic role of dysregulated apoptotic components in a myriad of conditions and disease models.

**Table 1:** Key publications in apoptosis systems modeling

Year	Author	Major findings	Ref.
2000	Fussenegger et al.	Theoretical study on mitochondrial permeabilization induced by BCL-2 family proteins	[127]
2004	Eissing et al.	Theoretical study analysing death receptor-induced apoptosis including bistability analysis	[128]
2004	Bentele et al.	ODE-based modelling of apoptosis signalling supported by quantitative Western blotting	[125]
2005	Stucki et al.	Theoretical study into mitochondrial cytochrome-c and SMAC release on caspase activation	[129]
2006	Rehm et al.	First study that combined single cell imaging and ODE modelling of caspase activation in response to mitochondrial permeabilization (MOMP)	[47]
2006	Bagci et al. & Legewie et al.	Theoretical studies that highlighted positive feedback loops which guarantee bistability subsequent to MOMP and co-operation in Apaf-1 oligomerisation	[130, 131]
2007	Chen et al.	Theoretical study on Bax-activation	[132]
2007	Lavrik et al.	Introduced robustness analysis into the field. Provided structural model of extrinsic apoptosis induced by CD95/Apo-1	[133]
2007	Eissing et al.	Theoretical studies to evaluate robustness of computational models against parameter variations	[134, 135]
2008	Albeck et al.	Combined study employing live cell imaging of caspase activation and MOMP, flow cytometry, immunoblotting and modelling during death receptor-induced apoptosis	[126]
2009	Zhang et al.	Theoretical study into how genotoxic stress proceeds to MOMP and caspase activation	[136]
2009	Chen et al. & Dussmann et al.	Application of stochastic models based on cellular automata (CA) to study Bax activation in mitochondrial membranes during apoptosis	[137, 138]
2009 & 2010	Rehm et al. & Huber et al.	Spatial signal propagation during apoptosis signalling including experimental testing and mathematical modelling using partial differential equations (PDE)	[123, 124]
2011	Aldridge et al.	Combined in silico and wet-lab analyses focussed on TRAIL-induced apoptosis	[139]
2011	Lau et al.	Identification of spatial and temporal aspects of apoptosis signalling	[140]
2012	Hector et al.	Clinical application of caspase modelling to predict recurrence in CRC	[13]
2012	Lee et al.	Combined study employing mathematical modelling and wet-lab validation identifying optimal treatment scheduling for apoptosis re-sensitization	[141]
2012	Gaudet et al.	Comprehensive assessment with sensitivity analyses of the impact of cell-to-cell deviations in protein concentrations on apoptosis dynamics	[142]
2012	Schleich et al.	Combined theoretical and wet-lab analyses into the role of apoptosis activation by caspase-8	[143]
2013	Lindner et al.	Use of BCL-2 systems analysis to predict patient response to chemotherapy in CRC	[18]
2013	Murphy et al.	Use of systems analysis to predict progression-free survival in GBM	[17]
2014	Kallenberger et al.	Combined in silico and wet-lab study into the regulation of apoptosis by caspase-8	[144]
2014 & 2015	Bertaux et al. & Roux et al.	Theoretical study investigating fractional killing in apoptosis	[145, 146]
2015 & 2016	Zhao et al. & Li et al.	Theoretical study into the role played by mutations in apoptosis signalling	[147, 148]
2017	Salvucci et al.	Large scale validation of caspase modelling as independent prognostic biomarker in CRC and refinement of the prognostic power of apoptosis systems models with machine learning	[19]
2017	Lindner et al.	Large scale validation of BCL-2 modelling as independent prognostic biomarker in CRC and analysis of apoptosis systems models in molecular subtypes of CRC	[20]
2018	Márquez-Jurado et al.	Study combining mathematical modelling with experimental microscopy data focussed on the role played by mitochondria in regulating apoptosis	[149]
2018	Hantusch et al.	Regulation of BCL-XL via Bax retrotranslocation	[150]

While individual proteins have limited prognostic power (reviewed in [151–153]) due to the complexity and signaling redundancy of the biological network under investigation, systems models have been shown to deliver powerful prognostic biomarkers. Application of such apoptosis systems models in the clinic requires the quantitative profiling of individual proteins (or proxy thereof such as mRNAs profiles) involved in apoptosis activation, followed by in silico simulation of apoptotic signaling based on the quantitative profiles of individual patients. The combination of patient specific apoptotic protein expression profiles and in silico simulations of apoptotic protein interactions is then able to deliver patient-specific predictions of apoptosis sensitivity. Recent studies by our group highlighted the applicability of the systems modelling approach, APOPTO-CELL, in predicting patient outcome, including in the GBM settings [17].

APOPTO-CELL is a mathematical model of caspases-dependent apoptosis validated against single cell microscopy experiments in HeLa cell [47]. The model describes the dynamic network of interactions of key proteins involved in the downstream apoptosis signaling with a set of ordinary differential equations. The model takes as input the concentration of key regulatory pro- and anti-apoptotic proteins, namely Apaf-1, Procaspase-3, Procaspase-9, Second Mitochondria-derived Activator of Caspases (SMAC) and X-linked inhibitor of apoptosis protein (XIAP). APOPTO-CELL outputs the temporal profile of cleaved Caspase-3 substrate (substrate cleavage, SC). Substrate cleavage represents the degree of caspases activation and thus the cell propensity to undergo apoptosis. APOPTO-CELL has been comprehensively tested in several cancer cell lines models [17, 105, 154] and mouse xenografts [105]. The application of APOPTO-CELL model for aggressive cancers such as the primary brain tumor, glioblastoma, was shown to provide an advantageous mean of predicting therapeutic efficacies based on individual patient expression profiles and is outlined as an example in this review [17].

#### Case study: application of APOPTO-CELL to GBM

When concentrations of the key proteins involved in the apoptosis machinery, Procaspase-3, Procaspase-9, SMAC and XIAP, were determined in GBM patient tumor resections, APOPTO-CELL was capable of stratifying patients by progression-free survival times (PFS) [17]. Since then, a greater number of patient tumor samples (*n* = 25 de novo and *n* = 21 previously published [17], totaling 31 samples (67%) isolated at initial-diagnosis and critically 15 (33%) isolated at tumor recurrence allowed for a more comprehensive analysis of the clinical applicability of APOPTO-CELL. Clinical characteristics of our in house GBM cohort including MGMT methylation and treatment(s) received before resection are highlighted in Table 2. In line with standard-of-care regimen [26], the newly-diagnosed tumor samples had not received any chemo/radio treatment prior to their surgical removal, while the recurrent tumor samples were obtained from patients who either had no follow-up treatment or who did receive chemo- and/or radiotherapy following their initial surgery. The clinical end-point for survival analysis was progression-free survival (PFS), defined as the time interval between surgical resection of the tumor (either newly-diagnosed or recurrent) and progression or loss to follow-up. Median PFS of 11.1 months (95% CI 8.4–16.8) and 5.8 months (95% CI 2.3–7.6) were observed for newly-diagnosed and recurrent tumors, respectively, in line with published literature [29, 30]. The histopathological and systems biology workflow is outlined below.

**Table 2:** Clinical baseline characteristics of GBM patient samples (*n* = 46)

	Newly-diagnosed (*n* = 31, 67%)	Recurrent (*n* = 15, 33%)
Age (median, range) [years]	57 (16–75)	53 (12–74)
Sex		
M	14 (45%)	10 (67%)
F	17 (55%)	5 (33%)
Location		
Left side	10 (32%)	3 (20%)
Right side	18 (58%)	10 (67%)
Other	2 (6%)	2 (13%)
Not available	1 (3%)	
MGMT promoter methylation		
Methylated	11 (35%)	6 (40%)
Unmethylated	14 (45%)	8 (53%)
Not available	6 (19%)	1 (7%)
Treatment		
None	31 (100%)	6 (40%)
TMZ + radiotherapy		7 (47%)
TMZ + radiotherapy + Avastin + Irinotecan		1 (7%)
TMZ + radiotherapy + Cilengitide		1 (7%)
PFS (median, 95%CI) [months]	11.1 (8.4–16.8)	5.8 (2.3–7.6)

*Abbreviations*: *TMZ* Temozolomide, *MGMT* O^6^-methylguanine-DNA methyltransferase

#### Protein profiling in patient tumor samples

**Fig. 1:**
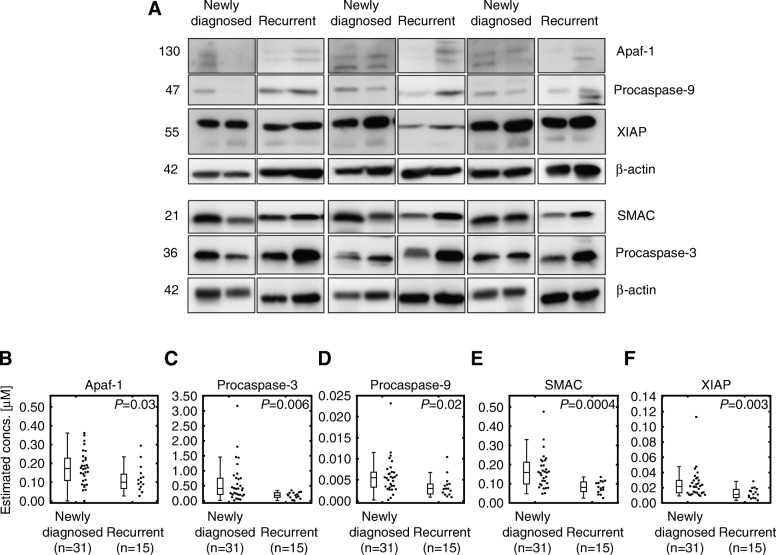
Newly-diagnosed tumors (*n* = 31) expressed higher protein concentrations of Apaf-1, Procaspase-3, Procaspase-9, SMAC and XIAP compared to specimens collected from recurrent patients (*n* = 15) in the GBM cohort. **a** Representative images of Western blot experiments. Each lane contains a unique patient tumor sample from newly-diagnosed or recurrent tumors as indicated. β-actin served as a loading control. **b**-**f** Normalized protein levels were converted to absolute concentrations (in μM, as required for inputting into APOPTO-CELL) by linear regression with known concentrations in HeLa cells [13, 17, 47]. Reference concentrations were previously determined in HeLa cell extracts with titrated concentrations of recombinant proteins [47]. Prior to pooling together protein quantifications for the de novo patients with those reported in [17], batch-effects in the measurements were removed. For each protein, the median concentration from the de novo newly-diagnosed samples was aligned to the median concentration measured in the newly-diagnosed specimens from [17]. Protein concentrations measured in tumor samples from de novo recurrent patients were also batch-corrected, but the scaling constants were computed based on median-aligning the newly-diagnosed samples only. Statistically significant differences between protein expression in newly-diagnosed vs. recurrent samples were examined by Mann-Whitney *U* tests

The expression of the proteins involved in caspase-mediated apoptosis, inputs to APOPTO-CELL, were determined by Western blotting in our in house GBM cohort (*n* = 46, Fig. 1a), normalized to β-actin and mapped to μM concentrations [13, 17, 47], (Fig. 1b-f). Heterogeneous protein levels can be clearly observed among patients, with Procaspase-3 (Fig. 1c) and Procaspase-9 (Fig. 1d) expressing the highest and lowest protein concentrations within tumor samples, respectively. Newly-diagnosed tumors were found to express higher concentrations of both pro- (Apaf-1, Procaspase-3, Procaspase-9 and SMAC) and anti-apoptotic (XIAP) proteins (*P* < 0.05, Mann–Whitney *U* test), (Fig. 1b-f).

**Fig. 2:**
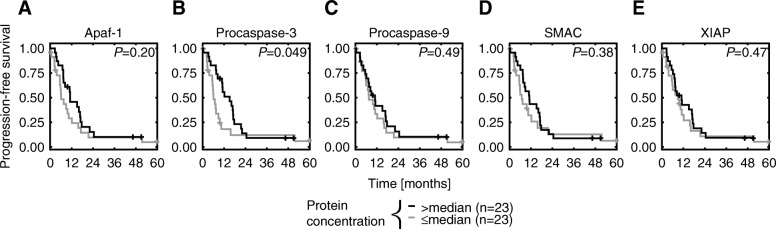
Assessment of the prognostic significance of single proteins regulating caspases-dependent apoptosis. **a**-**e** Kaplan-Meier estimates for Apaf-1 (**a**), Procaspase-9 (**c**), SMAC (**d**) and XIAP (**e**) showed no statistical significant differences in PFS curves among patients grouped by protein expression (>median vs. ≤median, in black and gray, respectively). Patients expressing higher concentrations of Procaspase-3 (>median) had longer PFS compared to those with low levels (≤median), (log-rank *P* = 0.049, **b**)

In line with more “classical” analyses, the prognostic significance of individual proteins was evaluated and benchmarked against APOPTO-CELL performance (Fig. 2). Kaplan-Meier estimates for patients grouped by protein expression (>median vs. ≤median) showed no difference in PFS for Apaf-1 (A), Procaspase-9 (C), SMAC (D) and XIAP (E), (Fig. 2). However, patients expressing low concentrations (≤median) of Procaspase-3 showed approximately a two-fold increased risk of progression (HR 1.91, 95% CI 0.99–3.69, *P* = 0.06) compared to those with high levels (Fig. 2b and Table 3). These findings indicate that single proteins have limited to no prognostic value in these settings.

**Table 3:** Cox proportional hazards regression models examining the prognostic value of clinical factors and signatures derived from single proteins and apoptosis modelling

Predictors	HR	95% CI	*P*
Age (continuous, *n* = 46)	1.02	1.00–1.05	0.06
Sex (ref. M, *n* = 24)			0.40
F (*n* = 22)	0.76	0.41–1.43	
Location (ref. left side, *n* = 13)			0.58
Right side (*n* = 28)	1.28	0.62–2.64	
Other (*n* = 4)	0.73	0.20–2.66	
History (ref. newly-diagnosed - no treatment, *n* = 31)			0.06
Recurrent - no treatment (*n* = 6)	1.86	0.69–5.01	
Recurrent - treatment (*n* = 9)	2.73	1.19–6.25	
MGMT promoter methylation (ref. methylated, *n* = 17)			0.52
Unmethylated (*n* = 22)	1.26	0.62–2.59	
Apaf-1 (ref. >median, *n* = 23)			0.20
≤ median (*n* = 23)	1.52	0.80–2.87	
Procaspase-3 (ref. >median, *n* = 23)			0.06
≤ median (*n* = 23)	1.91	0.99–3.69	
Procaspase-9 (ref. >median, *n* = 23)			0.49
≤ median (*n* = 23)	1.25	0.66–2.37	
SMAC (ref. >median, *n* = 23)			0.38
≤ median (*n* = 23)	1.34	0.70–2.55	
XIAP (ref. >median, *n* = 23)			0.47
≤ median (*n* = 23)	1.27	0.67–2.40	
Apoptosis susceptibility (ref. SC > 80%, *n* = 37)			0.001
SC ≤ 80% (*n* = 9)	5.02	2.04–12.33	
Adjusted apoptosis susceptibility (ref. SC > 80%, *n* = 37)^a^			0.006
SC ≤ 80% (*n* = 9)	4.40	1.59–12.14	

*P*-values determined by likelihood ratio tests

^a^Adjusted for age, history and MGMT promoter status

### Apoptosis susceptibility predicted by APOPTO-CELL is an independent prognostic marker of PFS

**Fig. 3:**
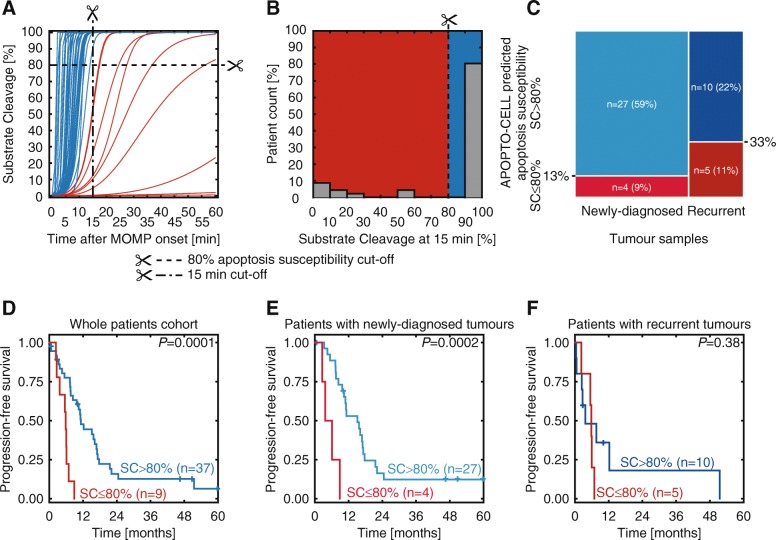
APOPTO-CELL model as a personalized risk assessment tool. **a** and **b** Patient-specific temporal profiles for substrate cleavage predicted by APOPTO-CELL (*n* = 46, **a**). The substrate cleavage reached at 15 min was deemed as the primary readout from the model simulations (**b**). Patients who did not cleave an amount of substrate of at least 80% were categorized as apoptosis-resistant (in red) whereas those above this threshold were classified as apoptosis-sensitive (in blue). **c** Association between apoptosis susceptibility predicted by APOPTO-CELL (SC ≤ 80% vs. SC > 80%, in red and blue, respectively) and type of tumor sample (newly-diagnosed and recurrent, light and dark shades, respectively). **d**-**f** Kaplan-Meier estimates of PFS in GBM patients categorized as apoptosis-resistant (*n* = 9, in red) or apoptosis-sensitive (*n* = 37 in blue) by APOPTO-CELL for the whole cohort (**d**) and stratified by type of tumor sample (newly-diagnosed and recurrent in **e** and **f**, respectively). *P*-values were determined by log-rank tests

APOPTO-CELL can predict the apoptotic propensity of tumor cells for each individual patient by initializing the model with protein concentrations assessed from their brain resections (Fig. 1b-f). Figure 3a depicts the model predictions (i.e. substrate cleavage time-courses) for each individual patient in our GBM cohort. Patients for whom substrate cleavage reached 80% within 15 min of simulation were classified as apoptosis-sensitive (in blue) whereas those who did not overcome this threshold were considered incapable of mounting apoptosis (in red). APOPTO-CELL predicted apoptosis deficiency in *n* = 9 (20%) patients (Fig. 3b). Exploratory analyses suggested a trend, albeit non-statistically significant (χ^2^ *P* = 0.10), whereby the fraction of patients predicted to be apoptosis-resistant (SC ≤ 80%) was greater in the recurrent tumors (*n* = 5 out of *n* = 15, 33%; dark red shade) than in newly-diagnosed cases (*n* = 4 out of *n* = 31, 12%; light red shade), (Fig. 3c). However, further analyses with greater number of patients and paired longitudinal samples are required to further investigate whether apoptosis deficiency worsens as tumors progress. Of note, statistically significant differences (*P* = 0.0001) in PFS were observed among patients categorized as apoptosis-sensitive vs. resistant (Fig. 3d). Patients with impairment in apoptosis (SC ≤ 80%, in red) showed approximately a five-fold increase in risk of progression (HR 5.02, 95% CI 2.04–12.33, likelihood ratio test *P* = 0.001) compared to participants predicted to be apoptosis-sensitive (SC > 80%, in blue), (Table 3). Within the patient group that harbored newly-diagnosed tumors, this observation was repeated and those patients with tumors that were predicted to mount an apoptotic response to treatment had significantly longer PFS, (*P* = 0.0002, log-rank test), (Fig. 3e). In contrast, we did not find a statistically significant association between apoptosis execution capability and PFS in the patient group (*n* = 15) that suffered recurrence (*P* = 0.38, log-rank test, Fig. 3f). Further studies in larger cohorts with a balanced set of newly-diagnosed and recurrent patients are required to investigate the relationship between apoptosis susceptibility and PFS.

Univariate Cox regression analyses examining the prognostic value of clinico-pathological characteristics, assessed routinely as part of the treatment decision plan, revealed limited utility in our in house cohort (Table 3). Critically, apoptosis susceptibility predicted by APOPTO-CELL remained an independent prognostic marker in multivariate analysis after adjusting for age, history of tumor samples and methylation status of the *MGMT* promoter (HR 4.40 95% CI 1.59–12.14, *P* = 0.006; Table 3).

Of note, functional alterations in caspases activation in the glioblastoma settings that may be uncovered by future research could be incorporated in APOPTO-CELL by revising the model skeleton (addition/deletion of reactions), by updating values for kinetic parameters and by modelling dynamics of additional species. This work could lead to a glioblastoma-specific APOPTO-CELL model with a revised panel of protein inputs to determine on a tumor-by-tumor basis and could indeed yield improved performance for APOPTO-CELL as prognostic marker for PFS in glioblastoma.

### Systems models as tools to inform treatment regimen

Figure 3 exemplifies how computational models can describe the state of a system (*apoptosis susceptibility*) for a given set of initial conditions (*protein concentrations at surgery*) on a patient-by-patient basis. A key advantage of these models over more “traditional” statistical approaches is the ability to predict what *would* happen upon perturbation of the system. Perturbations that mimic pharmacological interventions are of particular relevance for translational applications.

**Fig. 4:**
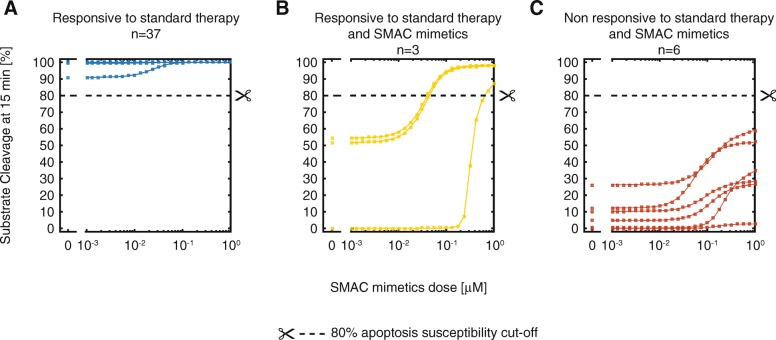
APOPTO-CELL can conduct in silico clinical trials for targeted apoptosis sensitization with SMAC mimetics. **a**-**c** Patient-specific dose-response curves simulated by APOPTO-CELL depicting the relationship between apoptosis susceptibility and pharmacological intervention. Apoptosis susceptibility is represented by the amount of simulated substrate cleavage reached at 15 min from the simulation start. Left hand-side of each plot before gap highlights basal apoptosis susceptibility (i.e. no administration of SMAC mimetics). Concentrations of SMAC mimetics tested in silico where selected to span the physiological doses administered in real-world clinical trials (1 nM - 1 μM). Patients were deemed “responsive to standard therapy” if classified as apoptosis-sensitive in simulations without any SMAC mimetics intervention (*n* = 37, **a**). Conversely, patients predicted to have apoptosis impairment in basal settings were deemed “responsive to only standard therapy and SMAC mimetics” (*n* = 3, **b**) or “non-responsive to standard therapy and SMAC mimetics” (*n* = 6, **c**) if administration of SMAC mimetics could induce (or not) re-sensitization, respectively

Figure 4 illustrates this point by simulating the impact that SMAC mimetics supplementation could have on apoptosis capabilities for each patient in our cohort. SMAC mimetics are compounds that, as the name suggest, mimic the effect of SMAC and thus induce apoptosis [155]. These small class of compounds are in phase II clinical trials and have so far shown to be effective in restoring apoptosis sensitivity in several cancers [105, 156–161].

Individual patient simulations were performed in basal conditions (no treatment) and with a physiological range of SMAC mimetics doses (1 nM - 1 μM). When comparing apoptosis susceptibility predicted by APOPTO-CELL among our GBM patients, three clusters of patients with distinct responses to SMAC mimetics were observed. Patients predicted as apoptosis-sensitive (SC > 80%) in basal simulations were considered “responsive to standard therapy” and targeted treatment was deemed unnecessary (Fig. 4a, *n* = 37). Patients predicted to remain apoptosis-resistant despite treatment with SMAC mimetics (Fig. 4c, *n* = 6) were considered “non-responsive to standard therapy and SMAC mimetics” and deemed unsuitable candidates for this class of targeted treatment. In contrast, patients categorized as apoptosis-resistant in basal simulations, but whose phenotype could be rescued with SMAC mimetics were considered “responsive to only standard therapy and SMAC mimetics” (Fig. 4b, *n* = 3). These patients are the optimal sub-population that should receive this treatment and should be prioritized for clinical trials for these compounds.

## Conclusions

Studies, such as those outlined above, showcase the applicability of systems models in the clinical workflow. Mathematical models predict not only the state of a system (tumor cell), but importantly they provide insights on *how* such phenotypic behaviour emerge, what the key components (proteins) and their interactions (wiring) are. Importantly, computational models provide a platform to put into context the impact that single components have when coupled in the system, epitomized by Aristotle’s quote “the whole is greater than the sum of its parts”. Importantly, computational models provide a tool to simulate ‘what-if’ scenarios such as up/downregulation of key components that could be targeted. In silico clinical trials, recently baptized phase *i* trials [162], aid in matching the “right drug to the right patient” [13, 18, 163]. A key idea is the shift from real-world clinical trial testing a single treatment option on many, mostly unselected, patients to *first* test in silico several treatment options, both approved or repurposed, for each individual patient. Importantly, mathematical models can also optimize the dosage and scheduling of the selected treatment [141, 162, 164–166].

Tailoring systems models to each patient tumour requires determining personalized inputs. Recent advances in proteomics [167–169] will provide the high throughput, spatially and temporal resolved patient-specific inputs that systems models such as APOPTO-CELL require. We anticipate that insights from multiple mathematical models (each describing key features of cancer cells) may be integrated via machine learning to shape the clinical management of GBM. It is critical for the adoption of systems models in translational settings that individual patient inputs can be measured rapidly and accurately with high-throughput techniques available in the clinic small portions of tumour samples. Moreover, the inputs to determine on a patient-by-patient basis should be minimized and optimized for the specific clinical application in hand [170]. While in research settings model inputs are typically quantified by Western blotting and/or reverse transcription polymerase chain reaction, alternative detection techniques need to be explored for clinical applications. Enzyme-linked immunosorbent assays [171, 172], multiplex immunoassays [173] or quantitative immunohistochemistry [174] are particularly appealing alternatives for protein-based inputs. In recent years, the use of gene expression as surrogate for protein levels has been put forward as transcriptomic assays (or reduced panels such as those provided by the Nanostring Technologies nCounter platform, https://www.nanostring.com/) are becoming more affordable and are now starting to be routinely integrated in the clinical portfolio. However, correlation between gene expression and protein levels may not be sufficiently high for all required inputs [175] and further studies are required to identify optimal combinations of genes that can serve as surrogate for protein expression.

Critical for any systems model is the validation of the predictions against experimental data. Recent advancements have made possible testing predictions from systems models in more physiological and clinically-relevant scenarios such those delivered by microfluidics chips [176], organoids [177], patient-derived xenografts [178] and tumor sponges [179].

With the establishment of more advanced and cost-effective technologies, often at single cell level, it is now possible to characterize different molecular layers (genome, epigenome, transcriptome and proteome), and to integrate with sophisticated data-driven systems biology approaches insights from spatially resolved longitudinal patient samples into a comprehensive atlas. Furthermore, international consortia such as GLIOTRAIN (Exploiting GLIOblastoma intractability to address European research TRAINing needs in translational brain tumour research, cancer systems medicine and integrative multi-omics, www.gliotrain.eu) and GLASS (Glioma Longitudinal AnalySiS Consortium, www.glass-consortium.org) bring together multi-disciplinary expertise to gather large scale patients-specific data to deliver a new generation of patient stratification tools for this aggressive form of cancer. We envisage that in a not-so-distant future, data- and hypothesis driven approaches from systems medicine will be routinely applied in the clinic and that “clinical decision support systems” will be developed to support reviewing of cases. Such systems will likely integrate machine learning algorithms to capture and analyse molecular and clinical data for each patient, and rank options for clinical management [180].

## Data Availability

Datasets and code to perform these analyses are publically available and archived at 10.5281/zenodo.3473419.

## Abbreviations

BCL-2: B-cell lymphoma 2
BCL-xL: B-cell lymphoma-extra large
CDKA: Cyclin-dependent kinase A
Chr5q: Chromosome 5q
CI: Confidence interval
CL: Classical molecular subtype
CSC: Cancer stem cell
CT: Computer tomography
EGFR: Epidermal growth factor receptor
FADD: Fas-associated protein with death domain
GBM: Glioblastoma multiforme
G-CIMP: CpG island methylator phenotype
GLASS: Glioma Longitudinal AnalySiS Consortium
GLIOTRAIN: Exploiting GLIOblastoma intractability to address European research TRAINing needs in translational brain tumour research, cancer systems medicine and integrative multi-omics
HR: Hazard ratio
IAP: Inhibitor of apoptosis
IDH: Isocitrate dehydrogenase
JNK: JUN N-terminal kinase
MES: Mesenchymal molecular subtype
MGMT: O^6^-methylguanine-DNA methyltransferase
MKK4: Dual specificity mitogen-activated protein kinase kinase 4
MKK7: Dual specificity mitogen-activated protein kinase kinase 7
ML: Machine learning
ML-PI: Hybrid machine learning (ML) and “Proliferation-Invasion” model (PI)
MRI: Magnetic resonance imaging
MYCN: N-Myc
NF1: Neurofibromin
NF-κB: Nuclear factor kappa-light-chain-enhancer of activated B cells
ODE: Ordinary differential equation
OS: Overall survival
P: *P*-value
PDGFRA: Platelet-derived growth factor receptor-A
PDX: Patient-derived xenograft
PFS: Progression-free survival
PI: “Proliferation-Invasion” model
PKB (or AKT): Protein kinase B
PN: Proneural molecular subtype
SC: Substrate cleavage
SMAC: Second Mitochondria-derived Activator of Caspases
TCGA: The Cancer Genome Atlas
TGF-β: Tumor growth factor-beta
TMZ: Temozolomide
XIAP: X-linked inhibitor of apoptosis protein
ZAK: Sterile alpha motif and leucine zipper containing kinase
